# A novel framework for classification of selection processes in epidemiological research

**DOI:** 10.1186/s12874-020-01015-w

**Published:** 2020-06-15

**Authors:** Jonas Björk, Anton Nilsson, Carl Bonander, Ulf Strömberg

**Affiliations:** 1 Division of Occupational and Environmental Medicine, Lund University, SE-221 85 Lund, Sweden; 2 Clinical Studies Sweden, Forum South, Skåne University Hospital, Lund, Sweden; 3 Centre for Economic Demography, Lund University, Lund, Sweden; 4 School of Public Health and Community Medicine, Institute of Medicine, Sahlgrenska Academy at University of Gothenburg, Göteborg, Sweden; 5 Department of Research and Development, Region Halland, Halmstad, Sweden

**Keywords:** Bias, Selection bias, Epidemiologic factors, Population characteristics, Public health

## Abstract

**Background:**

Selection and selection bias are terms that lack consistent definitions and have varying meaning and usage across disciplines. There is also confusion in current definitions between underlying mechanisms that lead to selection and their consequences. Consequences of selection on study validity must be judged on a case-by-case basis depending on research question, study design and analytical decisions. The overall aim of the study was to develop a simple but general framework for classifying various types of selection processes of relevance for epidemiological research.

**Methods:**

Several original articles from the epidemiological literature and from related areas of observational research were reviewed in search of examples of selection processes, used terminology and description of the underlying mechanisms.

**Results:**

We classified the identified selection processes in three dimensions: i) selection level (selection at the population level vs. study-specific selection), ii) type of mechanism (selection in exposure vs. selection in population composition), iii) timing of the selection (at exposure entry, during exposure/follow-up or post-outcome).

**Conclusions:**

Increased understanding of when, how, and why selection occur is an important step towards improved validity of epidemiological research.

## Background

*Selection* and *selection bias* are terms that still lack consistent definitions in epidemiology, and they also have varying meaning and usage in other scientific disciplines [1, 2]. This is a source of misunderstandings that may compromise validity, not the least in the interdisciplinary collaborations that are common within empirical research today. Most definitions of selection bias in epidemiology restrict the attention to sample selection in that selection is viewed as a bias-inducing phenomenon occurring at the study level, i.e. during design, enrolment, follow-up or analysis [3–5]. The focus on study-related issues when discussing selection bias implies that selection processes occurring at the population level, for example linked to migration, disease occurrence and mortality [6], are often overlooked among applied researchers. As an example of this narrow focus, it is sometimes claimed that selection bias is not an issue in cohort studies involving complete recruitment and follow-up [7].

There is also confusion in the literature between the underlying mechanisms that lead to selection and their consequences, i.e. the selection effects. As a result, working definitions of selection bias vary depending on whether consequences on internal or external validity are included [4, 8–10]. Unifactorial selection processes involving exposure or outcome but not both may distort measures of disease occurrence, for example outcome rates, risks and prevalence proportions, and may also hamper external validity of associational effect measures if the exposure effect is heterogeneous across population groups [10]. Bi- or multifactorial selection processes involving both exposure and outcome are generally necessary in order to distort the internal validity of causal effect measures [7, 8]. Selection effects must be judged on a case-by-case basis depending on the specific research question, study design and related analytical decisions. For this reason, presence or absence of selection effects cannot be determined generally for a cohort study that measures various exposures and outcomes [11]. The consequences of selection may even differ across analyses of a specific exposure-outcome association, for example in the main analysis versus in stratified or mediation analyses [2, 12].

Identifying and characterizing underlying selection processes when planning the study will aid the researcher in taking appropriate actions in design, data collection and analysis in order to enhance validity. The overall aim of this study was therefore to develop a simple but general framework for classifying various types of selection processes of relevance for epidemiological research. Examples of selection processes from the epidemiological literature are discussed and classified according to the proposed framework.

## Methods

### Notation and framework

We define a *selection process* as a process that results in a non-random split of a population or study cohort into two or more groups, either with respect to particular aspects such as exposure or health status, or more general aspects such as population membership or study eligibility. Standard causal diagrams (directed acyclic graphs, DAGs) are useful for depicting and defining the underlying mechanism behind different types of selection processes [8, 13]. Since the timing of the selection is crucial for judging its effect, it helps if the nodes are ordered chronologically from left to right in a causal diagram so that processes that are assumed to operate simultaneously are represented by nodes and arrows that are aligned vertically. We use subscripts for the nodes to indicate time, e.g. E_0_ represents exposure initiation in time window t = 0 and E_1_ exposure in some later time window t = 1.

### Literature review

In order to develop the framework, several highly cited original articles from the epidemiological literature as well as from related areas of observational research (economics, sociology and statistics) were reviewed in search of examples of selection processes, used terminology and description of the underlying mechanisms [2, 3, 8, 9, 14–22]. The reference lists of these articles, more recent publications and helpful reviewers led to identification of additional examples [1, 6, 10, 11, 23–25].

## Results

### A general framework in three dimensions

In our proposed framework, we classify selection processes in three main dimensions. Firstly, we distinguish between *selection processes at the population-level* occurring independently of study decisions and *study-specific selection processes* occurring only because of the study. Secondly, we distinguish between *selection in exposure*, i.e. selection processes that cause changes in exposure, and *selection in population composition*, i.e. processes that cause changes in the composition of the general population, source or study population. Thirdly, we organize the selection processes with respect to the timing relative the exposure and the outcome, for example *selection occurring at exposure entry*, *during exposure* (but prior to outcome) or *post-outcome*. These three dimensions are described in more detail in the following paragraphs.

### Population vs. study-specific selection processes

Population selection processes occur in general or source populations independently of study decisions, whereas study-specific selection processes occur only because of the study. Epidemiologists are often familiar with non-participation, self-selection into studies, losses to follow-up and other types of study-specific selection processes that can be serious concerns in empirical research. However, there are also selection processes that result in non-random groupings or changes in the composition of the underlying populations. These are continuously on-going at the population level irrespectively of whether they are subject to sampling in empirical studies [6]. Population selection occurs both within general and specific populations, such as specific patient populations. It includes phenomena that may lead to *confounding* in observational studies, i.e. confusion of effects or lack of exchangeability between exposed and unexposed with respect to background risks for the disease outcome [7]. The confounding resulting from population selection processes is sometimes apparent, such as differences in health determinants across groups in age, sex or socioeconomic characteristics, and therefore possible to adjust for in statistical analysis. However, population selection processes often lead to subtle differences across groups that are more difficult to account for, for example if personal ambitions make individuals seek higher education, if self-interest drives individuals to choose the occupation that produces the highest utility for them, if physicians select patients into treatments and when health conscious individuals select themselves into preventive screening programs. Additionally, population selection includes phenomena that are distinct from confounding, for instance if an index event must occur in order for someone to enter the eligible population [22]. As examples, disease progression can only be observed among people with the disease, and being unemployed is often a prerequisite for taking part in a job training program. Population selection may also act during exposure, for example through survival of the fittest and depletion of susceptibles over time [24]. A general characteristic of population selection effects is that they tend to persist also in “perfect” observational study settings, including register-based studies on entire populations [6].

### Selection in exposure vs selection in population composition

**Fig. 1:**
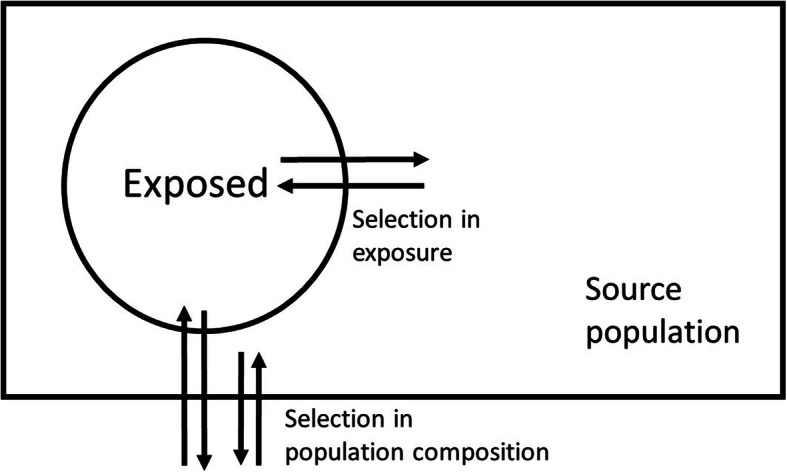
Selection in exposure vs. selection in population composition

**Fig. 2:**
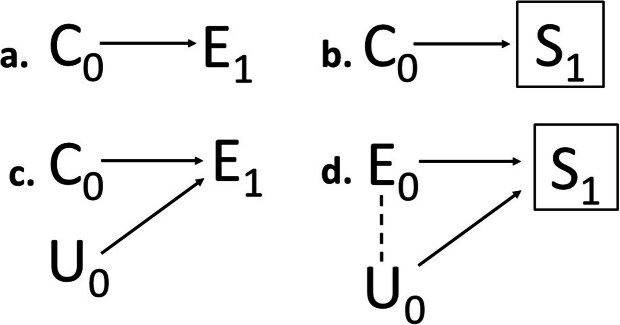
Fundamental selection mechanisms occurring at exposure entry at the population-level, illustrated by causal diagrams with subscripts of the nodes indicating time. **a.** Unifactorial selection in exposure (*E*_*1*_) **b.** Unifactorial selection in population composition (boxed *S*_*1*_) **c.** Multifactorial selection in exposure (*E*_*1*_) **d.** Multifactorial selection in population composition (boxed *S*_*1*_, induced inverse association is marked with a dashed line)

In the second dimension we make a mechanistic distinction between i) selection that causes changes in exposure (*selection in exposure*) and ii) selection through for example migration, disease events or deaths that causes changes in the composition of the population (*selection in population composition*; Fig. 1). Differences in mode of action between these two types of selection mechanisms, which can either be of unifactorial or bi/multifactorial origin, can be more formally depicted using causal diagrams (Fig. 2a-d).

#### Selection in exposure (unifactorial case)

Each arrow between two nodes in a causal diagram represents a direct causal effect, but also a directed selection process. To see why, consider the direct effect *C*_*0*_ → *E*_*1*_ in Fig. 2a, which here means that C-positivity (*C*_*0*_ = 1) increases the likelihood of becoming exposed (*E*_*1*_ = 1). As a consequence, C-positivity will be more common among exposed individuals and less common among unexposed individuals than in the total population. Thus, the direct causal link between *C* and *E* is a selection process that leads to two selected groups in the population, exposed and unexposed that have different compositions with respect to *C*. We will refer to any arrow that ends in initiated, continued or terminated exposure as *selection in exposure*. This selection process does not affect the boundaries of the population as such (i.e. it does not select in or out from the combined population of unexposed and exposed). In occupational epidemiology, *healthy worker hire effect* is a well-known example of selection in exposure that may lead to confounding bias in the estimated exposure effect on a disease outcome *D*, if healthy individuals (*C*_*0*_ = 1) in a population are more likely of becoming employed and thereby occupationally exposed (*E*_*1*_ = 1) [21]. If the origin of the selection *C* only causes *E* and not *D*, then confounding bias would not occur. However, external validity of associational measures can still be compromised by the causal link between *C* and *E* if there is heterogeneity in the *E* – *D* association across levels of *C* [10]. This would for example occur if individuals that are less susceptible to the exposure effect (e.g. more stress tolerant) are more prone to become exposed (by applying for positions with high job demands). The estimated *E* – *D* association would still be internally valid but would not generalize to the general population with a different distribution of susceptible individuals.

#### Selection in population composition (unifactorial case)

The other fundamental selection mechanism, *selection in population composition*, acts on the general, source or study population, for example by selecting individuals in or out from the population eligible for the exposure (e.g. only survivors until a certain age will be able to retire). It may also select individuals in or out from the population at risk during exposure (e.g. elevated preterm mortality in a drug addict population filters the population at risk of diseases typically occurring at older ages). As a result of this selection mechanism, there will be differences in the composition of subpopulations that are selected and filtered out, for example non-random differences between eligible and non-eligible or between those who remain at risk and those who do not. Selection in population composition is depicted in Fig. 2b, where the directed selection mechanism *C*_*0*_ → *S*_*1*_ here means that C-positivity (*C*_*0*_ = 1) will be more common those who remain in the population at risk (*S*_*1*_ = 1) than among those who do not (*S*_*1*_ = 0). At the population level, the boxed *S* represents what we refer to as *conditioning by nature*, which means that any causal action in this system downstream from this time window will only act on the selected population (*S*_*1*_ = 1). As with unifactorial selection in exposure, the change of the population composition with respect to *C* may compromise extern validity if the magnitude of the exposure effect depends on *C* [10].

#### Selection in exposure (multifactorial case)

Exposures are in reality often caused by several underlying selection mechanisms, either acting independently or in concert. Importantly, the origin of some these selections may be unknown. A selection process with two independent, directed selections into a common effect *E* is depicted in Fig. 2c, one originating from a known source *C* and one from an unknown source U. This combined selection process will generally induce associations between *C* and U within strata of *E* that are different from those of the underlying population. As an example, suppose heavy alcohol consumption (*E*) is caused by low socioeconomic status (*C*) but also by a genetically determined tolerance for alcohol that allows escalation of drinking, here assumed to be unknown (U). The induced inverse association within strata of alcohol consumption will imply that individuals with low socioeconomic status will have a worse tolerance on average than individuals with high socioeconomic status within the same stratum. Confounding bias in the estimated effect of *E* on a disease outcome *D* may occur also after adjustment for *C*, if the unknown exposure cause U is a direct or indirect cause of *D* [8]. Bias may occur even if U is only causing *D* in interaction with *C*. A hypothetical example is presented in Table 1, where the relative risk (RR) of *E* on *D* is constant (2.0) across strata of *C* and U. If the effect of *C* on *D* depends on U, then the causal effect of *E* can only be correctly identified in individuals where *C* is absent (so called partial exchangeability [26]). Bias will occur in analyses adjusted for *C* since exposed and unexposed individuals with the same level of *C* differ in U. Stratification on *C* may for this reason lead to false conclusions regarding the heterogeneity in the exposure effect.

**Table 1:** Data from a hypothetical cohort study with multifactorial selection in exposure (*E*) originating from *C* (known source) and *U* (unknown source; Fig. 2C). The relative effect of *E* on the disease outcome *D* is constant across strata of *C* and *U* (RR_*ED*_ = 2.0). *U* influences the effect of *C* on *D* (RR_*CD*_ = 1.5 when *U* = 0 but 4.0 when *U* = 1). Lack of adjustment for *U* therefore leads to bias in the estimated exposure effect also when *C* is adjusted for. Stratification for *C* may lead to false conclusions regarding the heterogeneity in the exposure effect

*U*	*C*	*E*	*D* = 1, n	*D* = 0, n	Risk	RR_*ED*_^1^	RR_*CD*_^2^
0	0	*E* = 1	20	80	20%		
		*E* = 0	90	810	10%	2.0	
	1	*E* = 1	90	210	30%		1.5
		*E* = 0	105	595	15%	2.0	1.5
1	0	*E* = 1	40	160	20%		
		*E* = 0	80	720	10%	2.0	
	1	*E* = 1	400	100	80%		4.0
		*E* = 0	200	300	40%	2.0	4.0
Totals		*E* = 1	550	550	50%		
		*E* = 0	475	2425	16%	2.32	
	0	*E* = 1	60	240	20%		
		*E* = 0	170	1530	10%	2.0	
	1	*E* = 1	490	310	61%		
		*E* = 0	305	895	25%	2.41	

^1^ Relative risk among exposed (*E* = 1) vs. unexposed (*E* = 0) across strata of *C* and *U*, overall and across strata of *C*

^2^ Relative risk among C-positive (*C* = 1) vs. C-negative (*C* = 0) across strata of *E* and *U*

#### Selection in population composition (multifactorial case)

Multifactorial selection processes also frequently operate on the population composition. An example is depicted in Fig. 2d, where the composition of an index population *S* (for example all cases of a certain disease) has an exposure *E* as a known origin but also an unknown origin U. Any subsequent causal action on disease progression can only be observed among people with the disease (*S* = 1), which in the causal diagram is represented by *S*. Such conditioning by nature on an index event necessary for entering the diseased population creates associations between the underlying determinants *E* and U that differ among people with and without the disease. This can lead to a bias commonly referred to as *index event bias*, or more generally *collider stratification bias* (or simply *collider bias*) in studies where the collider *S* (i.e. the event where the two directed selection mechanisms collide) constitutes the study population [16, 22]. As an example, smoking is a well-established risk factor for the development of rheumatoid arthritis (RA) [16]. Nevertheless, null or even inverse associations between smoking and disease progression among patients with RA have been observed. The antiinflammatory role of nicotine has been put forward as a possible explanation of the lower systemic inflammation and structural disease progression in current smokers with RA [27]. However, collider bias stemming from a selection mechanism of the type depicted in Fig. 2d is a compelling alternative explanation, as the necessary conditioning on the index event *S* (incident RA) induces spurious inverse associations between the exposure *E* (smoking) and other risk factors U that may also cause disease progression [16]. Another example of such paradoxical results that could be explained by collider bias is the apparent protective effect of obesity on mortality among end-stage renal disease patients [28].

### Timing of the selection – at exposure entry, during exposure or post-outcome

**Fig. 3:**
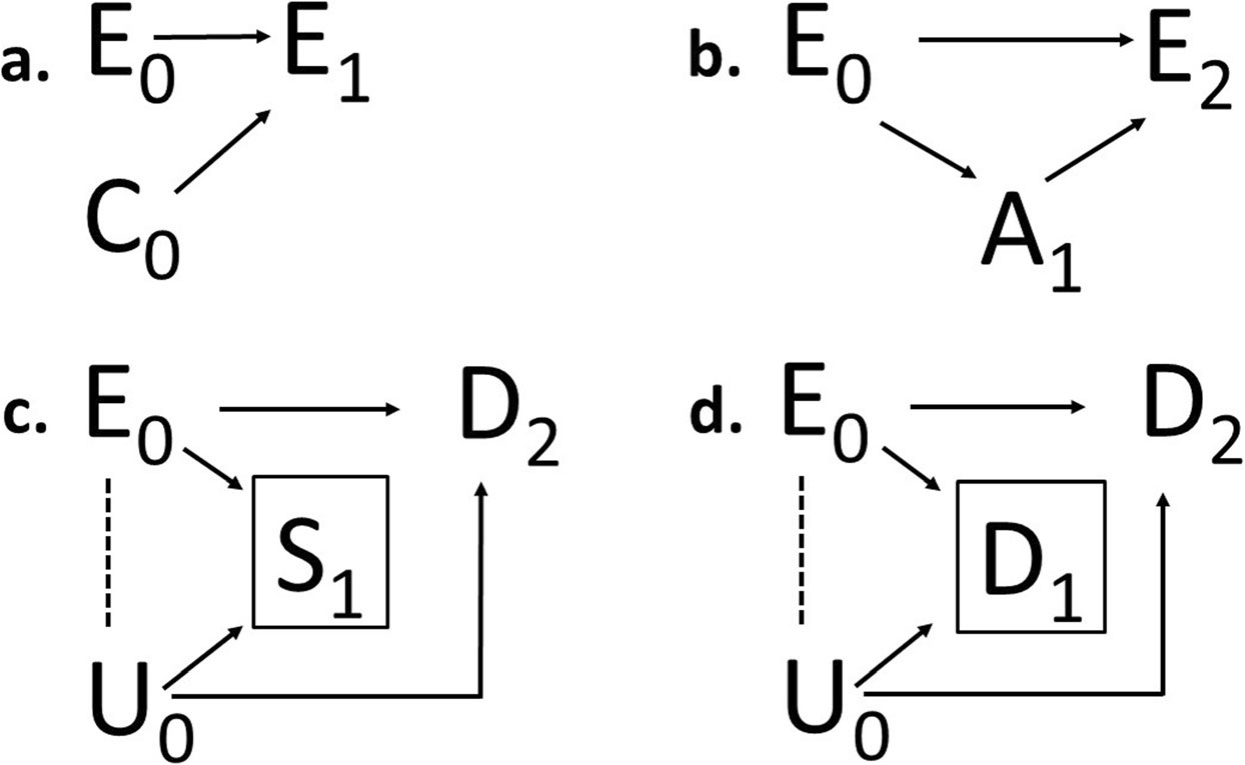
Selection during exposure at the population-level, illustrated by causal diagrams with subscripts of the nodes indicating time. **a.** A known determinant (*C*_*0*_) does not influence exposure initiation (*E*_*0*_) but exposure continuation (*E*_*1*_) **b.***E*_*0*_ causes a side effect (*A*_*1*_) that influences *E*_*2*_. **c.***E*_*0*_ and an unknown determinant (*U*_*0*_) cause an index or competing event (boxed S_1_) that precludes the disease outcome (*D*_*2*_) **d.***E*_*0*_ and *U*_*0*_ cause an early disease event (boxed *D*_*1*_) that leads to depletion of susceptibles that precludes later disease events (*D*_*2*_) to occur. Induced inverse associations are marked with dashed lines

**Fig. 4:**
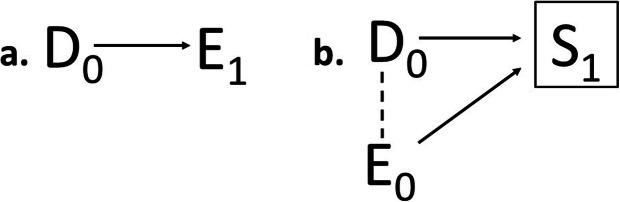
Selection post-outcome at the population-level, illustrated by causal diagrams with subscripts of the nodes indicating time. **a.** Selection in exposure (*E*_*1*_) caused by disease outcome (*D*_*0*_) **b.** Selection in population composition (boxed *S*_*1*_) caused by disease outcome (*D*_*0*_) and exposure (*E*_*0*_). Induced inverse associations are marked with dashed lines

Figure 2a-d all represent selection mechanisms occurring at exposure entry, prior to any exposure or treatment effect in the downstream population. At the study-level, selection occurring at exposure entry corresponds to non-random sample selection at study entry in surveys and baseline selection before follow-up starts in cohort studies [6, 29]. Selection may also act during exposure (Fig. 3a-d), and affect continuation and termination of exposure or follow-up. Selection during exposure would for example occur if individuals less susceptible to side effects are more prone to stay exposed or continue with a treatment (see additional example in next section). Similarly, selection post-outcome would occur if subclinical symptoms lead to loss to follow-up in a study, or if a disease outcome lead to changes in exposure. The latter would be an example of *reversed causality*, meaning that the outcome causes exposure changes rather than the opposite (Fig. 4a). This situation would lead to bias unless data allow separation of the timing of the outcome events and exposure changes. Selection post-outcome would also lead to bias if the composition of the general, source or study population is dependent both on disease outcome and exposure (Fig. 4b). A particular example is selective participation and nonresponse bias in studies conditioning on variables affected by the outcome and exposure [2].

### Selection processes at the population-level – additional examples

We now illustrate how the proposed framework with three dimensions (selection level, type of mechanism and timing of the selection) can be used to classify selection processes commonly described in the epidemiological literature (Table 2). In this section we focus on population selection, whereas next section covers study-specific selection processes.

**Table 2:** Commonly described selection processes at the population-level in the epidemiological literature classified according to the proposed framework

Level	Type of mechanism	Timing of the selection		
		*At exposure entry*	*During exposure*	*Post-outcome*
(1) *Population- level*	(1.1) *Selection in exposure*	(1.1.1) Self-selection Confounding by indication Healthy worker hire effect	(1.1.2) Side effects causing exposure changes Healthy worker survivor effect	(1.1.3) Subclinical symptoms causing exposure changes
	(1.2) *Selection in population composition*	(1.2.1) Index event Self-selection	(1.2.2) Competing event Depletion of susceptibles	(1.2.3) Berkson’s fallacy
		*At study entry*	*During follow-up*	*Post-outcome*
(2) *Study-specific*	(2.1) *Selection in exposure*	(2.1.1) Self-selection	(2.1.2) Non-compliance^1^	(2.1.3) Non-compliance^1^
	(2.2) Selection in population composition	(2.2.1) Restriction of source population Study base definition Self-selection Healthy volunteer effect	(2.2.2) Loss to follow-up	(2.2.3) Loss to follow-up Selective participation in a case-control study

^1^Non-compliance with treatment (exposure) administration specified in the study protocol

#### Selection in exposure – at exposure entry, during exposure or post-outcome

*Self-selection* into a screening program for breast-cancer implemented in the general population may result in important differences between exposed (i.e. those attending the screening) and unexposed women (those who do not attend), including social, demographic, and health factors that can independently influence outcomes [30]. Self-selection at the population-level (Table 2, cell 1.1.1) has several context-specific synonyms such as *confounding by indication* in clinical research, which occurs when the clinical indication for selecting a particular treatment (for example severity of the illness) also affects the outcome [31], and the abovementioned *healthy worker hire effect* in occupational epidemiology [21].

Selection in exposure at the population-level may also occur during exposure (Table 2, cell 1.1.2), even when exposure initiation was not subject to selection. As an example, there was no apparent socioeconomic gradient (*C*) in the propensity to start smoking (*E*_*0*_) among young adults in the 1950’s [32]. However, the propensity to continue smoking (represented by *E*_*1*_ in Fig. 3a) despite growing evidence of serious health hazards exhibited a clear inverse socioeconomic gradient [33]. The selection from exposure initiation to exposure continuation can be mediated by a side effect or an adverse event caused by the exposure. This situation would for example occur if initial smokers (*E*_*0*_ = 1 in Fig. 3b) who experience bad cough (*A*_*1*_ = 1) are more likely to quit smoking than others. Because of this selection, continuing smokers (*E*_*2*_ = 1) would then have experienced bad cough to a lesser degree than those who have given up smoking (*E*_*2*_ = 0). *Healthy worker survivor effect* is a similar selection process, which implies that employees who can tolerate the physical or psychosocial working conditions are more prone to stay in the workplace and thereby remain occupationally exposed [21].

Selection may also cause changes in exposure post-outcome (Table 2, cell 1.1.3), for example if a certain exposure may relieve subclinical symptoms. As an example, it has been suggested that smoking in schizophrenia may begin in the prodromal phase of the disorder [34].

#### Selection in population composition – at exposure entry, during exposure or post-outcome

As already discussed in relation to Fig. 2d, selection in population composition occurring prior to exposure entry often implies that an *index event* (for example obtaining a certain age, becoming unemployed or falling ill [22]) must have occurred in order to make an individual eligible for the exposure (Table 2, cell 1.2.1). Self-selection may also affect population composition, for example if people motivated to exercise and eat well choose to live in neighborhoods that support this lifestyle [35]. Changes in population composition may also occur during exposure (Table 2, cell 1.2.2). Figure 3c represents a situation where an exposure *E* causes an adverse event *S* (with death as an extreme example) that precludes continued stay in the population at risk with respect to the disease outcome of interest *D*. Such mutually exclusive outcomes are commonly referred to as *competing event (competing risk*) [15]. Continued causal actions beyond a certain time window can only occur if the adverse event has not occurred until then. This implies that the exposure in the population at risk will gradually become more and more inversely related to other determinants of the adverse event (represented by U in Fig. 3c). As an example, suppose we want to study the effect of sedentary lifestyle (*E*) on the risk for dementia (*Y*) at older ages. Thus, people will have to have survived to older ages in order to become part of the exposed population at risk. This necessary conditioning on survival (*S* = 1) implies that surviving individuals with sedentary lifestyle can be expected to have a more favorable risk profile with respect to other determinants (U) of survival than surviving individuals with a physically active lifestyle. Biased effect estimates would be obtained if these determinants are also related to the risk of dementia and are not accounted for.

*Depletion of susceptibles*, which is depicted in Fig. 3d, is a selection process that is similar to the competing event situation (Fig. 3c), in that the continued exposure *E* causes selection gradually in population composition (Table 2, cell 1.2.2). However, here the outcome at a later time point (represented by *D*_*2*_ in Fig. 3d) does not get competition from other outcomes but from the same outcome at earlier time points (*D*_*1*_). Thus early events in the outcome of interest caused by the exposure may lead to depletion of susceptibles in the population over time [24]. As an example, an effect of smoking on a particular disease outcome may seemingly decrease over time, or even change direction, as early disease events “eat” of the causal components required for the disease to manifest at older ages. It has been suggested that the hazardous effect of smoking on mortality may disappear for ages 85 and above [36]. However, an alternative explanation is that the harmful effect of smoking is disguised by depletion of susceptibles, i.e. smokers among the oldest are likely to be a more selected group of survivors than non-smokers at the same age with respect to other determinants of survival. Bias will occur in the estimated smoking effect unless susceptibility can be measured and accounted for.

Selection processes may also alter the composition of the population post-outcome (Table 2, cell 1.2.3). A well-known example is *Berkson’s fallacy*, where the population of patients who come to the hospital is structurally different from patients with the same disease who for various reasons do not come [18]. The selected population may be dissimilar from the unselected population with respect to a single determinant of the selection, but also with respect to associations between different determinants (Fig. 4b with *S* as selected population). Post-outcome selection of the Berkson type may for example affect validity in studies of malformations in live births [37], as malformations often increase the risk of miscarriages and these are often impossible to observe.

### Study-specific selection processes – additional examples

#### Selection in exposure – at study entry, during follow-up, or post-outcome

Study-specific selection in exposure may occur at study entry in studies of medical or social interventions, where exposure or treatment is not randomized but assigned by the investigator or chosen by the study person (e.g. as a prerequisite for participation). Such *self-selection* (Table 2, cell 2.1.1) may lead to bias, and can also occur post-randomization in randomized studies, in particular in trials where treatment assignment cannot be blinded and participants are free to refuse treatment [25]. *Non-compliance* with the treatment protocol caused by side effects (during follow-up; Table 2, cell 2.1.2) or by subclinical symptoms (post outcome; Table 2, cell 2.1.3) may also affect the validity of interventional studies.

#### Selection in population composition – at study entry, during follow-up or post-outcome

Selection in population composition at study entry may either be ‘intentional’ or ‘unintentional’ from the researcher’s perspective [6]. Intentional selection occurs based on criteria established by the researcher, for example when restricting the source population for a cohort study or defining the study base for a case-control study (Table 2, cell 2.2.1). Intentional selection may have consequences on validity. As an example, randomized clinical trials and observational cohort studies on effects of hormone-replacement therapy on coronary heart disease have yielded conflicting results. A major reason for the differences was the inappropriate definition of the source population in several of the cohort studies [19]. Long-term current users of estrogen/progestin were included at baseline, which led to selection of individuals less susceptible to adverse effects and with biased treatment effects as a consequence. Unintentional *self-selection* affecting population composition at study entry occurs when there are differences between the source population (those who are eligible to participate) and the study population (those who actually participate). A particular example is the *healthy volunteer effect* (i.e. participants being more healthy than non-participants [15]), which may hamper the possibilities to generalize results beyond the study sample. Both intentional and unintentional baseline selection (at study entry) may in cohort studies lead to collider bias of the type depicted in Fig. 3c, for example if study participation (*S*) is caused both by the exposure (*E*) of interest and another outcome risk factor (U) [6].

*Loss to follow-up* in cohort studies before or after outcome has occurred (Table 2, cells 2.2.2 and 2.2.3) can be critical if outcome ascertainment is dependent on continued study participation (for example by an additional visit to the study center) [38], but is generally of less concern if outcomes are ascertained by health care registers [39]. Selection occurring post-outcome is of particular concern in case-control studies if enrolment is done retrospectively after ascertainment of case/control-status [40].

## Discussion

The important distinction between population and study selection is presently lacking in most working definitions of selection bias. Early work by Kleinbaum et al. used the term selection bias to denote a distortion in the estimate of effect resulting from the manner in which subjects are selected into the study population [3]. Similarly, Delgado-Rodriguez & Llorca defined selection bias as the error introduced when the study population does not represent the target population [15]. However, these restrictive definitions to a large extent exclude selection occurring at the population level, which may therefore fly under the radar in the mind of applied researchers when interpreting their results. Furthermore, in the commonly used working definition of confounding (confusion of effects [41]), the reason why exposed and unexposed individuals differ with respect to a third variable is not specified. Knowledge about underlying selection processes are essential for the choice of adequate study design, appropriate statistical methods and correct analytical decisions [9]. We therefore find it inappropriate to have restrictive or unspecific definitions of central validity concepts in epidemiology that do not naturally lead investigators to think carefully about potential reasons why the populations of exposed and unexposed individuals may lack comparability, either already at study entry or evolving gradually during follow-up.

Our proposed framework in three main dimensions may bridge some of the gaps between previous work on selection in different scientific disciplines that have either viewed selection as a phenomenon that occurs prior to exposure [14, 42], or as a phenomenon that occurs downstream the exposure from selecting on a collider [8, 13]. The Cochrane handbook for systematic reviews of interventions defines selection bias in clinical observational studies as “systematic differences between baseline characteristics of the groups that arise from self-selection of treatments, physician-directed selection of treatments, or association of treatment assignments with demographic, clinical, or social characteristics” [42]. Self-selection is thus a keyword for this definition, i.e. the selection occurs at exposure or treatment entry. The baseline characteristics for which the differences are observed are not necessarily the true underlying factors responsible for the selection, but are likely to be associated with them. A related issue is therefore to what extent the resulting selection effects can be accounted for by the observed differences in baseline characteristics.

Selection into social programs was the specific selection mechanism considered in a seminal paper by Heckman et al. [14]. The authors made a decomposition of the resulting selection effect into three components: 1) systematic differences in covariate patterns among treated and untreated, 2) lack of sufficient support (overlap) for certain covariate patterns, 3) selection bias, precisely defined. With this decomposition, confounding in the sense that the treatment effect is confused with that of the observed covariates would be due to differences and lack of support in covariate pattern (component 1 and 2), whereas any differences not attributable to the observed covariates would be the remaining selection effect (component 3).

Hernan et al. [8] have proposed a now widespread distinction between confounding and selection bias based on causal diagrams: confounding occurs when there exist common causes of exposure and outcome, while selection bias results from conditioning on common effects of exposure and outcome. The underlying mechanism in the Hernan selection (also denoted *endogenous selection bias* in subsequent work [2]) is different from the selection bias defined by Heckman [14], where selection is into exposure rather than occurring as a consequence of the exposure. In our proposed framework, we argue that selection processes are ubiquitous, i.e. present in all the arrows of a causal diagram, and common causes (confounding), conditioning on common effects (endogenous selection) and Heckman selection are thus specific examples of selection processes that can be characterized by our proposed framework.

Consistent with the third dimension of our proposed framework, Elwert et al. [2] characterize the endogenous selection of the Hernan type further depending on the timing of the conditioning, i.e. selection acting on pre-treatment, post-treatment, outcome or post-outcome variables. Our framework allows for distinction between selection processes and the potential consequences of selection, which are closely linked to the context and study objectives. As an example, suppose there is a selection process affecting a particular population at risk at exposure entry in a unifactorial manner (for example related only to current health status represented by *C*_*0*_ in Fig. 2b). An investigator whose main interest is to report a valid estimate of the relative exposure effect may be less concerned about this selection than another investigator whose objective is to report risk differences (or number needed to harm), assess the etiologic fraction, or generalize the findings to the unselected population.

Some study limitations should be noted. Our study was not based on a systematic review of the literature and may thus not have detected all relevant aspects of selection in observational research. Two additional aspects, uni- vs. multifactorial selection [10, 21] and intentional vs. unintentional selection [6], were identified and described but not included as separate dimensions in the proposed framework. Another limitation was that reviewing different analytical approaches to correct for selection effects was outside the scope of the study. The effects of selection can be adjusted for using various methods depending on the research question, underlying selection mechanisms and causal structures [1, 8, 11, 43]. Only a few notes are made here. Firstly, the reason why general study participation predictors (such as age and sex) should be accounted for in the analysis can be baseline collider bias (selection in population composition at study entry) rather than classical confounding (selection in exposure) [6]. Secondly, inverse probability weighting (IPW) is a preferable method to standard regression adjustment for factors causing selection in order to reduce bias from self-selection at exposure or study entry and from loss to follow-up [11]. The reason for this is that regression adjustment may for some causal structures open up new backdoor paths from exposure to outcome by conditioning on other colliders [8]. Thirdly, handling threats to external validity due to selection typically requires auxiliary data from non-participants, or a random sample of the target population, and that IPW methods are applied [1]. Using auxiliary data is an attractive approach to correct for selection effects in for example the Nordic countries, where individual-level data from population and health care registers can be linked to cohort members and target population members through personal identification numbers [39, 43].

## Conclusions

Based on a literature review, we have proposed a framework for classification of selection processes as occurring either at the population or study level. We further distinguished between selection processes that cause changes in exposure and selection processes that cause changes in population composition. We additionally organized the selection processes with respect to the timing relative the exposure and the outcome. Our proposed framework can be used to identify and classify selection processes that may lead to lack of comparability of exposed and unexposed populations or decrease study validity in other ways, either already at exposure initiation or gradually evolving during exposure. We expect the use of this framework to increase awareness among applied researchers of how selection processes may influence, or even jeopardize, validity of epidemiological research.

## Data Availability

Not applicable.

## Abbreviations

DAG: Directed acyclic graph
IPW: Inverse probability weighting
RR: Relative risk
